# Poverty, Human Development, and the Role of eHealth

**DOI:** 10.2196/jmir.9.4.e34

**Published:** 2007-10-22

**Authors:** Gunther Eysenbach

**Affiliations:** ^1^Centre for Global eHealth InnovationUniversity Health NetworkToronto ONCanada

**Keywords:** Human Development, Poverty, Medical Informatics, Developing Countries

## Global Theme Issue on Poverty and Human Development

In this issue, the Journal of Medical Internet Research (JMIR) publishes five papers [1-5] which are part of the Global Theme Issue on Poverty and Human Development, organized by the Council of Science Editors [6]. Today, on October 22^nd^, 2007 (a few days after the United Nations’ World Poverty Day on October 17^th^), more than 235 science journals throughout the world simultaneously publish papers on this topic of worldwide interest - to raise awareness, stimulate interest, and stimulate research into poverty and human development. This is an international collaboration with journals from developed and developing countries. Some journals dedicate an entire issue to this subject, others publish a few papers, and still others only publish an editorial. Almost 1000 papers will be published in this Global Theme Issue.

Amazingly, JMIR is the only Medline-indexed health informatics journal participating in this initiative.

This is surprising, because the potential of ehealth to improve life expectancy, literacy, education, and standard of living (all dimensions of “human development”) is significant, as illustrated by some papers in this issue.

At the same time it is exactly poverty (of individuals and communities), lack of literacy and deficits in education that often prevents or limits the use and access to information and information technology in underdeveloped settings. Seven years ago, I proposed the now widely used term “inverse information law” [7] to characterize this dilemma: *Access to information is often most difficult for those who need it most.* The “inverse information law” was formulated in analogy to Tudor Hart’s “inverse care law”, which describes that access to health care is often most difficult for those populations who need it most.

This paradox is also echoed in some of the papers we publish in this issue, but the papers published here also offer inspiration for how information technology may be used effectively in resource-poor settings.

Hamish Fraser and colleagues have been deeply involved in deploying information systems in developing countries. In their paper [1], which is a combination of a classical literature review enriched with personal experiences, they draw attention to the fact that the impact of devastating diseases such as AIDS and tuberculosis could be dramatically reduced by employing information and communication technologies. In resource poor settings, where often access to even the most basic health care is lacking, electronic health records rank perhaps lowest in the priority list of funding agencies and other decision-makers. However, even in these environments, medicine remains an information intensive business, and the very high rates of loss to follow-up in many African HIV treatment programs, with the best program retaining 85% of patients and the worst retaining 46% [8] is essentially an information management problem, where information and communication technology can help.

Another clear example of the role of ehealth in addressing human development issues are telehealth programs, bridging gaps in health care between developed and underdeveloped regions. Patterson and colleagues present a telehealth program between one of the most developed countries in the world - Australia - and war-torn Middle Eastern countries such as Iraq and Afghanistan [2], and Valenzuela and colleagues present a project within Colombia [5]. For such teleconsultation programs to work, the underlying technology must be simple – email store-and-forward systems, perhaps combined with inexpensive digital cameras, fulfil this requirement. These projects have the potential to positively influence the education and life expectancy components of human development. In Iraq, as Patterson and colleagues write, 13 years of international sanctions and three devastating wars in the past 25 years have made it difficult for local health professionals to keep up to date. Patterson and colleagues show that a doctor-to-doctor consultation service is feasible and generally greeted with enthusiasm [2]. However, they also ran into the “inverse information law” paradox, as access to the Internet limits this type of service. As an aside one should also note that it is not always the expert in the developed world who can learn from this information exchange. As one quote from a Director of Health in Iraq suggests, doctors and nurses are experts in “managing with very little”, and are probably more versed in infectious diseases which occur in their local settings than international experts. Clearly, the case can be made that the knowledge transfer through telemedicine is not unidirectional.

The teleconsultation project by Valenzuela and colleagues [5] is different from the Australian project, in that the target group for teleadvice are directly consumers rather than health care providers, and in that the project aims to bridge the health services divide *within* a less developed country (in 2006, Colombia was ranked 70th out of 177 countries, according to Human Development Index data). Again, we find evidence for the inverse information law, as only 0.2% of requests came from remote areas, where access to health services is suboptimal. This is similar to previous observations from a similar project, where we observed that “we did not encounter any patient where physical disabilities, remoteness, or living in a medically underserved area appeared to be the reason for seeking information on the Internet and teleadvice.” [9].

Poverty and digital divide remain an issue in developed countries as well. The work of Kontos and colleagues [3] provides some fascinating insights into the mechanisms of how poverty in the United States can negatively impact the ability to use information technologies. Among other barriers, people with lower socioeconomic status cite time constraints as one important factor, together with the fact that they have to compete with their kids for the (sometimes only) computer in the household. As facilitators, the role of social networks should be explored further.

Finally, the paper by Hassan Masum and Peter Singer on visual dashboards [4] reminds us that information and communication technology can also help in policy priority setting, not only through the visualization and dissemination of information, but also in the information gathering phase, by employing the Internet as a platform for public engagement.

Authors with relevant research who missed our call for papers are encouraged to submit their work to any regular JMIR issue – we remain very interested in this topic, particularly on how to improve the lives of those who have the least. After all, the open access model of this journal is also an example of how information technology can make the world a more equitable and better place, facilitating knowledge transfer to those who would otherwise not be able to access scientific and technical information.

Gunther Eysenbach,

*Editor, JMIR*

### Addendum

(Note added on 13 Dec 2007) The following paper was added to the Poverty Theme Issue after publication of this editorial: McNeill LH, Puleo E, Bennett GG, Emmons KM. Exploring Social Contextual Correlates of Computer Ownership and Frequency of Use Among Urban, Low-Income, Public Housing Adult Residents. J Med Internet Res 2007;9(4):e35, URL: http://www.jmir.org/2007/4/e35/
